# Predicting mortality and no‐reflow in STEMI patients using epicardial adipose tissue

**DOI:** 10.1002/clc.23692

**Published:** 2021-07-13

**Authors:** Amr Mohamed

**Affiliations:** ^1^ Department of Internal Medicine Rochester General Hospital Rochester New York USA; ^2^ Department of Cardiology Ain Shams University Cairo Egypt

**Keywords:** epicardial adipose tissue, no‐reflow, STEMI

## Abstract

**Background:**

Acute myocardial infarction is a leading cause of morbidity and mortality worldwide. It occurs when irreversible myocardial cell damage or death occurs. ST‐segment elevation myocardial infarction (STEMI) is the most serious presentation of atherosclerotic coronary artery disease. STEMI results from the occlusion of a major coronary artery. Primary percutaneous coronary intervention (PCI) is the preferred reperfusion strategy. It should be performed by an experienced team within the shortest time possible from the first medical contact.

**Hypothesis:**

Increased mortality risk and no‐reflow in STEMI patients with epicardial adipose tissue thickness (EAT) more than 5 mm compared to STEMI patients with EAT less than 5 mm.

**Methods:**

This study was conducted on 113 patients who presented to the cardiology department of Ain Shams university hospital with the first STEMI and underwent primary PCI. Medical treatment for STEMI was given to all subjects as per the guidelines. All patients underwent an echocardiographic evaluation of epicardial adipose tissue and left ventricular ejection fraction. Patients were divided into two groups using epicardial adipose tissue thickness of 5 mm as a cutoff point; this number was derived from the ROC curve. Group I: Included patients with EAT thickness less than 5 mm, including 44 patients (38.9%). Group II: Included patients with EAT thickness greater than 5 mm, including 69 patients (61.1%).

**Results:**

The current study showed that epicardial fat thickness significantly correlated with the no‐reflow phenomenon in the cath lab and overall prognosis in patients with STEMI.

**Conclusion:**

Increased EAT thickness may be an independent predictor of the no‐reflow phenomenon and mortality. Therefore, our study emphasizes that EAT thickness measured by echocardiography may provide additional and substantial information on the risk of no‐reflow and mortality in STEMI patients.

## 1 INTRODUCTION

ST‐elevation myocardial infarction (STEMI) is the most serious presentation of atherosclerotic coronary artery disease. Primary percutaneous coronary intervention (PCI) is considered the preferred reperfusion modality for patients presenting with STEMI.^1^ Microvascular occlusion may manifest angiographically as reduced flow in the patent upstream epicardial arteries, a situation that is termed “no‐reflow” or “slow flow.”

No‐reflow is also defined as less than TIMI III flow in the absence of abrupt closure, high‐grade stenosis, or flow‐limiting dissection. This event is associated with increased infarct size and reduced recovery of ventricular function; furthermore, this phenomenon is also linked to ventricular arrhythmia, early congestive heart failure, or even cardiac rupture.^2^

There are data on the relationship between epicardial adipose tissue thickness as a microvascular disease marker and no‐reflow.^3^ Adipose tissue affects cardiovascular physiology via the release of active adipokines in a paracrine and endocrine manner.^4^ Peri‐coronary EAT released leptin promotes endothelial dysfunction, leading to coronary atherosclerosis progression and plaque vulnerability; in addition, EAT produces pro‐inflammatory and anti‐inflammatory cytokines and could affect the adjacent coronary arteries by these cytokines.^5^

Epicardial fat is the true visceral fat located within proximity of the myocardium, between the pericardium visceral and parietal layer, and it shares the same blood supply as the adjacent myocardium. It also shows paracrine functions. This is the risky fat that is metabolically active.^6^

This article aims to demonstrate the relationship between epicardial adipose tissue thickness and no‐reflow phenomena in patients undergoing primary PCI.

### 1.1 Study methods

Informed consent had been obtained from all the study participants.

The research committee had approved the study at Ain Shams University. We did not experiment with any new treatment on patients, and all patients received the standard of care of treatment according to the most recent guidelines.

This study is an exploratory pilot study conducted on patients presented to Ain Shams University Hospitals with acute ST‐segment elevation myocardial infarction and underwent primary PCI. The relationship between EAT thickness detected by 2D echocardiography and no‐reflow phenomena detected by coronary angiography and GRACE score will be studied. The echocardiography operators were blinded to the result of the coronary angiogram.

We included all patients with a definite diagnosis of acute STEMI With no age or sex predilection presenting in the first 12 h of chest pain onset.

We excluded patients presenting later than 12 h, patients with a previous history of CABG, STEMI patients who did not undergo primary PCI, Mechanical complications of MI as acute mitral regurgitation and ventricular septal rupture, Patients with chronic kidney disease.

All patients had written informed consent and were subjected to complete history taking, 12 lead surface EKG on admission and 90 min after primary PCI, then every 8 h in the first 48 h then daily till discharge. In addition, complete blood work including serum creatinine and serial follow‐up of cardiac biomarkers (CK‐CKMB) every 8 h in the first 24 h, then once daily till discharge.

All patients underwent echocardiography within 48 h of hospital admission and with machine‐integrated ECG recording. The patients were lying in the left lateral decubitus position. We used a Vivid S6 machine with an M4S probe. Standard images were obtained in the parasternal (long‐ and short‐axis) and apical (2–3 and 4‐chamber images) views. Standard 2D and color Doppler data, triggered to the QRS complex, were saved in cine‐loop format. M‐mode, 2D, TDI as well as pulsed and continuous Doppler flow across the different heart valves in all the standard views were done with particular emphasis on LV end‐diastolic diameter and LV end‐systolic diameter using short‐axis parasternal window at the level of papillary muscles and LVEF calculated by M‐mode. EAT was assessed on the right ventricle's free wall from parasternal long‐axis view using the aortic annulus as an anatomical reference in at least two locations parasternal longitudinal axis view using the mean of three consecutive beats. A cutoff value of 5 mm for EAT will be used.

Regarding the Intervention procedure details: All patients will receive 600 mg of loading clopidogrel and 300 mg of aspirin. All patients underwent diagnostic coronary angiography followed by handling the culprit by primary PCI. Assessment of post‐stenting TIMI flow will be done.

We used the GRACE score to predict mortality. The score will be calculated for each patient and correlated with EAT thickness and no‐reflow. The score variables include age, heart rate (HR), systolic blood pressure, creatinine level, Killip class of heart failure, cardiac arrest at admission, ST‐segment deviation, and cardiac enzymes.

During hospitalization: aspirin, clopidogrel, anticoagulant, statins, ACEIs, nitrates, and beta‐blockers were given to the patient if not contraindicated.

## 2 STATISTICAL ANALYSIS

Data analysis was done using a statistical program for social science (SPSS) version 16 as follows: Description of quantitative variables as mean, *SD*, and range. Description of qualitative variables as number and percentage. Unpaired t‐test was used to compare quantitative variables in parametric data (*SD* < 50% mean). Comparison between groups as regards qualitative variables was done by using the chi‐square test. Fisher exact test was used instead of chi‐square when one expected cell is less than 5. A one‐way ANOVA (analysis of variance) test was used to compare more than two groups regarding quantitative variables. Spearman, correlation coefficient test was used to rank variables versus each other positively or inversely. The receiver operator characteristic (ROC) curve was used to determine the best cutoff value and validity of certain variables. *p* value >.05 nonsignificant (NS), *p* < .05 significant (S), *p* < .001 highly significant (HS).

## 3 RESULTS

The study population comprised 113 patients with STEMI who underwent primary PCI at Ain Shams University hospitals from 2018 to 2019. About 50% of the patient had anterior STEMI, and the other half had inferior STEMI.

**FIGURE 1:**
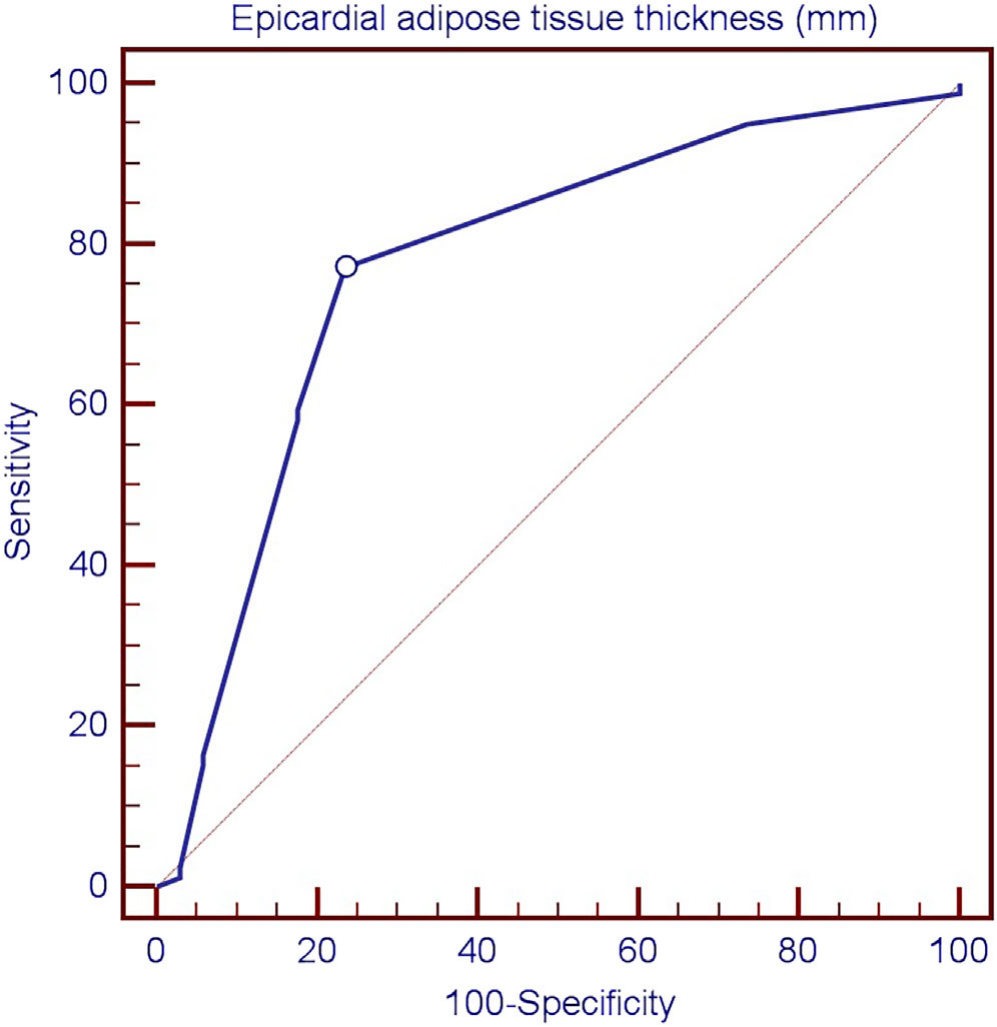
Receiver operator characteristic (ROC) curve for predicting mortality

Patients were divided into two groups using epicardial adipose tissue thickness of 5 mm as a cutoff point. The 5 mm cutoff point was derived from the ROC (Receiver Operating Characteristics) curve for the total calculated score between EAT thickness and GRACE mortality risk, which showed that the best cutoff point for the occurrence of mortality was greater than 5 mm EAT thickness with a sensitivity of 77%, and a specificity of 76%, positive predictive value 88.4%, and a negative predictive value of 59.1% as demonstrated in figure 1.

AUC (Area Under Curve) is considered as a rough guide for classifying the accuracy of a diagnostic test, an area of 1 represents a perfect test while an area of 0.5 represents a worthless test, the AUC for EAT ROC curve was 0.774, and this indicates a fair test result.

- Group I: Included patients with EAT thickness less than 5 mm, including 44 patients (38.9%).
- Group II: Included patients with EAT thickness greater than 5mm, including 69 patients (61.1%).

Both groups were age‐matched (55.3 ± 9.57) years in group I versus (57.12 ± 8.62) years in group II, and gender‐matched (77.3%) males in group I versus (84.1%) males in group II. There was no statistically significant difference between both groups regarding age and gender, with a *p* value of 0.297 and 0.366, respectively, as shown in Table A in the supplementary material section.

There was a statistically significant difference between the two groups regarding DM and dyslipidemia with a *p* value of .048 and .008, respectively, while no statistically significant difference regarding hypertension, smoking, and PVD with a *p* value of 0.223, 0.3, and 0.208, respectively as shown in Table B in the supplementary material section.

There was a statistically significant difference between the two groups regarding GRACE score with a *p* value of .0005, while no statistically significant difference regarding BMI, ECG, pain to door time with a *p* value of 0.849, 0.279, and 0.343, respectively, as shown in Table 1.

**TABLE 1:** Represents the relevant clinical data between the two groups

Variable	Group I		Group II	Independent *t* test	
	*N* = 44		*N* = 69	t/X^2^	*p* value
BMI	Mean ± SD	28.20 ± 2.65	28.30 ± 2.74	−0.191	0.849
ECG	Anterior	19 (43.2%)	37 (53.6%)	1.172	0.279
	Non anterior	25 (56.8%)	32 (46.4%)		
GRACE mortality risk	Mean ± *SD*	2.05 ± 1.08	3.47 ± 1.87	−4.568	.0005
Chest pain (pain to door)	Mean ± *SD*	6.52 ± 2.52	7.01 ± 2.77	−0.953	0.343

**FIGURE 2:**
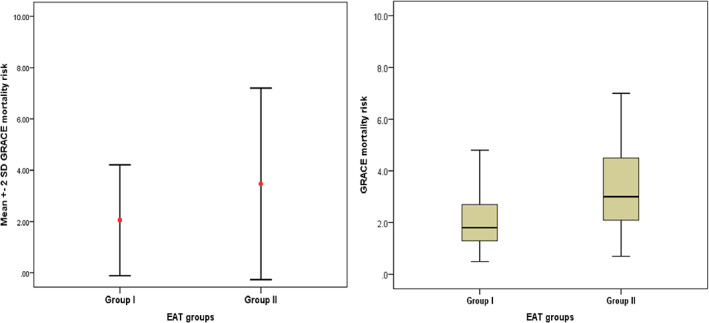
GRACE mortality risk between both groups, on the right graph values, are given as mean ± 2SD, on the left graph values are provided as a median, range, and IQR (interquartile range) group II had GRACE mortality risk of mean 3.47 ± 1.87, while group I had GRACE mortality risk of mean 2.05 ± 1

**FIGURE 3:**
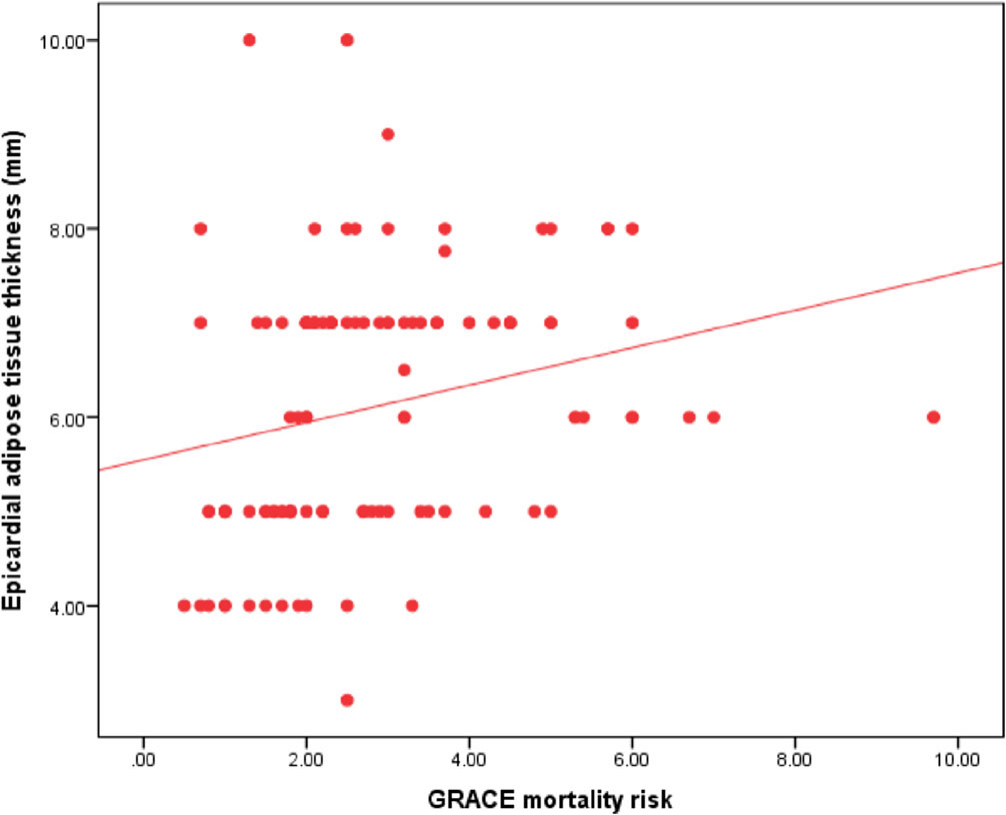
Shows the correlation between the GRACE mortality risk and epicardial adipose tissue thickness (EAT) thickness

Figure 2 will help to illustrate further the GRACE mortality risk difference between the two groups. Group II had a GRACE mortality risk mean of 3.47 ± 1.87, while group I had a GRACE mortality risk mean of 2.05 ± 1. There is also a positive correlation between epicardial adipose tissue thickness and mortality risk using the GRACE score from the above Figure 3.

Regarding coronary vessel involvement in both groups. Single vessel disease was found in 86.4% of patients in group I versus 24.6% of patients in group II. as regards multi‐vessel disease, it was found in 13.6% of patients in group I versus 75.4% of patients in group II. Thus, there is a statistically significant difference between the two groups with a *p* value of .0005, as shown in Table 2.

**TABLE 2:** Comparing the two groups regarding the presence of multi‐vessel disease and TIMI flow

Variable		Group I		Group II		Chi‐square test	
		No	%	No	%	X^2^	*p* value
Other vessels	Single vessel disease	38	86.4%	17	24.6%	51.123	.0005
	Multi‐vessel disease (2 or 3 vessel DS)	6	13.6%	52	75.4%		
TIMI flow	TIMI = 3	44	100.0%	7	10.1%	87.600	.0005
	TIMI < 3	0	0.0%	62	89.9%		

Abbreviation: TIMI, thrombolysis in myocardial infarction.

Regarding the no‐reflow phenomenon incidence between the two groups. Group II had 62 cases where the angiographic flow was less than TIMI III (89.9%), while group I had no cases with angiographic no‐reflow. There is a statistically significant difference between the two groups with a *p* value of .0005, as shown in Table 2.

## 4 DISCUSSION

In the present study, patients with EAT thickness > 5 mm had more no‐reflow phenomenon compared to patients with EAT thickness < 5 mm. Furthermore, our study showed that high EAT thickness was an independent predictor of the no‐reflow phenomenon.

EAT was found to be associated with metabolic syndrome. Our results were similar to Iacobellis et al.^7^ regarding diabetes mellitus and dyslipidemia with a *p* value of .048 and .008, respectively. In addition, Iacobellis mentioned that higher epicardial fat thickness is associated with impaired fasting glucose in nondiabetic men and women with a *p* value of <.001.

Unlike Iacobellis et al.,^8^ his study showed that body mass index and waist circumference (*p* = .01) were strongly correlated with EAT thickness. Our study failed to establish a relationship between body mass index and EAT thickness. This was mainly attributed to the small sample size and the strict exclusion criteria. We did not include chronically ischemic patients, NST acute coronary syndrome (ACS) patients, chronic kidney disease patients, and CABG patients. In addition, most of our population were first‐time presenters with acute STEMI. No one in our study population had a history of prior myocardial infarction or PCI.

Eroglu et al.'s^9^ showed that EAT thickness was increased in hypertensive patients compared to normotensive controls (6.3 ± 1.7 mm vs. 5.3 ± 1.6 mm; *p* < .001). However, our study did not show a relationship between hypertension and increased EAT thickness. This was mainly attributed to the small sample size. In addition, most of Eroglu et al.'s patients had uncontrolled hypertension. In comparison, most of our patients had controlled blood pressure.

Reperfusion injury, micro‐embolization of plaque or thrombus from the primary lesion site, and obstruction of the capillaries due to inflammation mediated by neutrophil activation are the proven mechanisms that play a pivotal role in no‐reflow.^10^, ^11^, ^12^ It is also known that metabolic factors such as insulin resistance, hyperglycemia, and hyperlipidemia are mainly caused by visceral obesity. Hemostatic factors such as increased thrombus burden induced by these metabolic risk factors significantly increase the risk of no‐reflow phenomenon.^13^, ^14^ It is also known that metabolic syndrome is a predictor of ST‐segment resolution after primary PCI in patients with STEMI; lack of ST‐segment resolution is a marker of no‐reflow.^15^ In keeping with these previous studies, our findings revealed that EAT thickness and the other indicators of visceral obesity were significantly associated with no‐reflow.

Microscopically, EAT is composed of adipocytes, stromovascular, inflammatory, and immune cells. Both adipocytes and tissue macrophages produce adipokines and cytokines. EAT produces pro‐inflammatory and anti‐inflammatory cytokines and could affect the adjacent coronary arteries by these cytokines.^16^ Adiponectin expression in EAT was detected to be lower in patients with coronary artery disease.^17^ According to these data, EAT may also be associated with no‐reflow because of being involved in the inflammation process. In previous studies, the anterior location of STEMI was associated with the no‐reflow phenomenon.^18^ In our study, there was no difference between the anterior and inferior location of STEMI as regard no‐reflow incidence.

Our study agrees with Erlogu et al. and Chaowalit et al.^19^, ^20^ that EAT thickness is closely associated with the presence of multi‐vessel disease with a *p* value of .0005. No‐reflow was also more evident in patients with multi‐vessel disease as compared to patients with a single‐vessel disease with a *p* value of .0005, and this agrees with the results by Cenko, Edina et al.^21^

Our study showed that epicardial adipose tissue thickness detected by echocardiography correlated with short‐term prognosis in patients with STEMI. We hypothesized that the short‐term prognosis after ACS correlated with epicardial fat thickness. We agreed with Park et al.^22^ in their study, where they found a good correlation between epicardial adipose tissue and MACE occurrence. This was also mentioned by Ndrepepa et al.^23^ They stated that in patients with STEMI treated by primary PCI, the no‐reflow phenomenon is a strong predictor of 5‐year mortality. No‐reflow phenomenon after PCI provides prognostic information that is independent of and beyond that provided by infarct size, and since increased epicardial fat is strongly related to no‐reflow, therefore, he indirectly agrees with our study. Although we could not make a direct comparison of this association in our study, we used GRACE mortality risk, which provided an indirect but strong positive correlation between mortality risks, increased EAT thickness, and no‐reflow; some mechanisms may be evoked to explain this correlation. We could assume that epicardial fat leads to long‐lasting exposure to inflammatory stimuli. Increased cytokines may contribute to a pro‐atherogenic and pro‐thrombogenic environment on the coronary arteries.

To the best of our knowledge, this is the first study using GRACE in conjunction with EAT thickness in primary PCI patients for assessing mortality risk.

## 5 CONCLUSION

Increased EAT thickness may be an independent predictor of the no‐reflow phenomenon. Therefore, our study emphasizes that EAT thickness determined on echocardiography may provide additional and substantial information on the risk of no‐reflow in STEMI patients treated with primary PCI.

## CONFLICT OF INTEREST

The author declares there is no potential conflicts of interest.

## Supporting information


**Table A** Comparison between cases group I and group II regarding age and gender.
**Table B.** Comparing both groups for the prevalence of risk factors of CAD: Click here for additional data file.

## Data Availability

The data that support the findings of this study are available from the corresponding author upon reasonable request.
