# Supplementary material for: Predicting mortality and no‐reflow in STEMI patients using epicardial adipose tissue
Source: Clin Cardiol. 2021 Jul 13;44(10):1371–6. doi: 10.1002/clc.23692 (PMC8495074; doi:10.1002/clc.23692)
Supplement: Supplementary file 1 — Table A Comparison between cases group I and group II regarding age and gender. Table B. Comparing both groups for the prevalence of risk factors of CAD: [file CLC-44-1371-s001.docx]

**Table (A):** Comparison between cases group I and group II regarding age and gender.

| Variable | | **Group I** | **Group I**I | **Independent t-test** | |
| --- | --- | --- | --- | --- | --- |
|  |  | **No. = 44** | **No. = 69** | **t/X²*** | **P-value** |
| Age | Mean ± SD | 55.30 ± 9.57 | 57.12 ± 8.62 | -1.049 | 0.297 |
|  | Range | 35 – 80 | 32 – 76 |  |  |
| Gender | Female | 10 (22.7%) | 11 (15.9%) | 0.818 | 0.366 |
|  | Male | 34 (77.3%) | 58 (84.1%) |  |  |

**Table (B):** Comparing both groups for the prevalence of risk factors of CAD:

| **Variable** | | **Group I (n=69)** | **Group II (n=44)** | **Chi-Square test** | |
| --- | --- | --- | --- | --- | --- |
|  |  |  |  | **X²** | **P-value** |
| DM % | No | 68.2% | 49.3% | 3.910 | 0.048 |
|  | Yes | 31.8% | 50.7% |  |  |
| Hypertension% | No | 52.3% | 40.6% | 1.483 | 0.223 |
|  | Yes | 47.7% | 59.4% |  |  |
| Dyslipidemia% | No | 29.5% | 10.1% | 6.942 | 0.008 |
|  | Yes | 70.5% | 89.9% |  |  |
| PVD % | No | 97.7% | 100.0% | 1.582 | 0.208 |
|  | Yes | 2.3% | 0.0% |  |  |
| Smoking status% | Drug addict | (2.3%) | (0.0%) | 2.408 | 0.300* |
|  | Heavy smoker | (65.9%) | (75.4%) |  |  |
|  | No | (31.8%) | (24.6%) |  |  |

*: Chi-Square test
